# The European quality of care pathways (EQCP) study on the impact of care pathways on interprofessional teamwork in an acute hospital setting: study protocol: for a cluster randomised controlled trial and evaluation of implementation processes

**DOI:** 10.1186/1748-5908-7-47

**Published:** 2012-05-18

**Authors:** Svin Deneckere, Martin Euwema, Cathy Lodewijckx, Massimiliano Panella, Walter Sermeus, Kris Vanhaecht

**Affiliations:** 1 Public Health School, Faculty of Medicine, KU Leuven, Kapucijnenvoer, Leuven, Belgium; 2 Faculty of Psychology and Educational Sciences, KU Leuven, Tiensestraat, Leuven, Belgium; 3 European Pathway Associa0074ion, Kapucijnenvoer, Leuven, Belgium; 4 Faculty of Medicine, Amedeo Avogadro University of Eastern Piedmont, Via Duomo, Vercelli, Italy; 5 Western Norway Research Network on Integrated Healthcare Helse Fonna, Haugesund, Norway

**Keywords:** Study protocol, Care pathways, Interprofessional teamwork, Cluster randomised controlled trial, Process evaluations

## Abstract

**Background:**

Although care pathways are often said to promote teamwork, high-level evidence that supports this statement is lacking. Furthermore, knowledge on conditions and facilitators for successful pathway implementation is scarce. The objective of the European Quality of Care Pathway (EQCP) study is therefore to study the impact of care pathways on interprofessional teamwork and to build up understanding on the implementation process.

**Methods/design:**

An international post-test-only cluster Randomised Controlled Trial (cRCT), combined with process evaluations, will be performed in Belgium, Ireland, Italy, and Portugal. Teams caring for proximal femur fracture (PFF) patients and patients hospitalized with an exacerbation of chronic obstructive pulmonary disease (COPD) will be randomised into an intervention and control group. The intervention group will implement a care pathway for PFF or COPD containing three active components: a formative evaluation of the actual teams’ performance, a set of evidence-based key interventions, and a training in care pathway-development. The control group will provide usual care. A set of team input, process and output indicators will be used as effect measures. The main outcome indicator will be relational coordination. Next to these, process measures during and after pathway development will be used to evaluate the implementation processes. In total, 132 teams have agreed to participate, of which 68 were randomly assigned to the intervention group and 64 to the control group. Based on power analysis, a sample of 475 team members per arm is required. To analyze results, multilevel analysis will be performed.

**Discussion:**

Results from our study will enhance understanding on the active components of care pathways. Through this, preferred implementation strategies can be defined.

**Trail registration:**

NCT01435538

## Background

Interprofessional teamwork is essential for the delivery of high quality healthcare. The report of the Institute of Medicine (IOM) *‘To Err is Human: Building a Safer Health System’* showed that 3% to 4% of patients hospitalized in the United States (US) were harmed by the care they received. The IOM concluded that poor teamwork and communication was a contributing factor in more than one-half of medical errors [1,2]. Classen *et al.* indicate that adverse events rates may be even ten times higher than previously measured [3]. Teamwork in healthcare is defined as a dynamic process involving two or more health professionals with complementary backgrounds and skills, sharing common health goals, and exercising concerted physical and mental effort in assessing, planning, or evaluating patient care [4]. In industries such as aviation and automobile manufacturing, the value of high-performance teams has long been realized [5]. The Agency for Healthcare and Research Quality (AHRQ) has argued to learn from the experience in these industries and implement various interventions that could improve teamwork as a strategy for enhancing patient safety and reducing medical errors [6]. A meta-analysis on team-training interventions across different settings reported that they account for approximately 20% of the variance in team performance [7]. A RAND report that reviewed 16 healthcare studies found empirical evidence supporting the relationship between teamwork and patient outcomes [8].

One of the interventions that can promote teamwork in healthcare is the implementation of care pathways [9-12]. Care pathways are widely used quality improvement initiatives for organising and reorganising care processes [13]. Compared with other care coordination interventions, such as the chronic care model or integrated care, care pathways are most effective for standardizing low complexity and low uncertainty care processes [14]. Uncertainty implies in the context of care coordination that the course of disease or treatment of a particular patient is unpredictable. The use of care pathways supports healthcare teams in implementing evidence-based key interventions and reduce clinical variations in everyday practice [15]. Furthermore, they are high-performing work systems that improve organisational performance by strengthening relationships and coordination among team members [10,11]. Considering different types of team training, as defined by the AHRQ [6], we see care pathway development as a behaviour change intervention containing a mix of cross-training, self-correction, and team-building exercises. Through care pathway development, roles and tasks of team members are traded, interprofessional relations are improved, feedback on performance is provided, short- and long-term team goals are set, evidence-based practice increases, an overall team vision and shared mental model is built up, and high-performance teams can be established.

Looking at most systematic reviews on pathway-effectiveness studies, the primarily focus is on how they affect hospital costs, patient outcomes, and length of stay [16-18]. The way they affect teamwork is seldom documented. This could be explained because the main goal of care pathways is in fact to improve patient processes and outcomes. Improved teamwork is then merely seen as one of the important mediators and facilitators to achieve that goal [19,20]. However, as Thomas *et al.* state, quality improvement initiatives as care pathways, although not always explicitly targeted, could actually improve teamwork and even have better results for patients than other team training interventions that focus solely on teamwork [21]. A recent systematic review did find a positive, but cautious, indication that a relationship between care pathways and teamwork does exist [12]. Although evidence was of rather low quality, most frequently positive effects of care pathways were found on staff knowledge, interprofessional documentation, team communication, and team relations. The review also identified some negative effects of care pathways, of which most importantly were increased workload and emerging team conflicts through care pathway implementation. Furthermore, as mentioned in their commentary on the review by Salas *et al.*, good teamwork is not only about standardized communication protocols [22]. Attention in care pathways should be given to create the ability to adapt, innovate, and react efficiently and effectively when the situation calls for it. The review concluded that the absence of high-level evidence is due to the lack of high-quality designs and the failure to use validated team indicators in pathway-effectiveness studies.

The European Pathway Association (E-P-A) defines a care pathway as ‘a *complex intervention* for the mutual decision making and organisation of care for a well-defined group of patients during a well-defined period’ [23]. As complex interventions, they are built up from a number of components that may act both independently and interdependently [24]. Although they are difficult to specify, these interacting components seem essential to the proper functioning of the intervention. The more it is difficult to exactly define the active components of an intervention, and how these interrelate, the more it is likely that the intervention is a complex one [24,25]. Care pathways seem to be at the higher end of the complexity spectrum. According to an international survey on use of care pathways, possible active components are increased evidence-based care, improved coordination of the care process, improved communication with the patient, improved teamwork, improved follow-up and increased efficiency [13]. Nonetheless, strong evidence on active components and causal pathways is lacking. Furthermore, knowledge on the process of pathway implementation is sparse and variations in how organisations go about the implementation process are large [19]. Multiple behavioural and organisational components will influence the extent to which they can be successfully integrated into everyday healthcare practice. Evaluating and implementing care pathways therefore requires understanding on how and in what circumstances they work by exploring the context in which they are implemented and the interrelating mechanisms that define their success [26,27].

### Objectives

The primary objective of the European Quality of Care Pathways (EQCP) study is to evaluate the impact of care pathways on interprofessional teamwork in healthcare teams in an acute hospital setting. A secondary goal is to build up knowledge on the active components of care pathways and on the conditions under which they can be most effective through evaluation of the implementation processes.

## Methods

### Setting

The EQCP study is an international multicentre research project launched by the European Pathway Association (E-P-A) (http://www.E-P-A.org), an international not-for-profit association. The E-P-A is collaborating with the Centre for Health Services and Nursing Research of the Faculty of Medicine of the Catholic University Leuven (Belgium) and the School of Public Health of the Amedeo Avogadro University of Eastern Piedmont (Italy) who take the scientific lead in this study. As explained in more detail in Vanhaecht *et al.*[23], the overall project consists of three parts: a trial focusing on the impact of a care pathway for exacerbation of chronic obstructive pulmonary disease (COPD-exacerbation) on patient processes and outcomes; a trial focusing on the impact of a care pathway for proximal femur fracture (PFF) on patient processes and outcomes; and a trial we are discussing here focusing on the impact of care pathways on interprofessional teamwork in which both COPD-exacerbation and PFF-clinical teams are included. The study is being performed in four countries: Belgium, Ireland, Italy and Portugal. In each country, a research centre is coordinating the project based on an international agreed protocol and a national coordinator is appointed. Furthermore, each participating team is asked to appoint a study coordinator as local facilitator for the study.

### Study design

**Figure 1:**
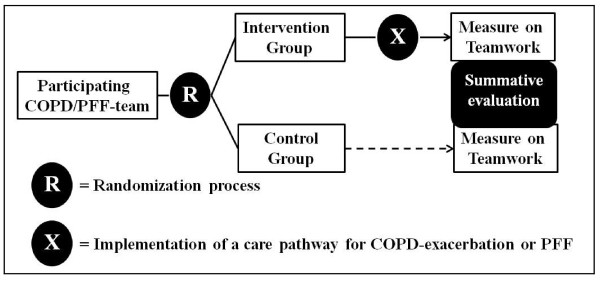
Study design of the EQCP-trial on teamwork.

To evaluate the effect of the care pathway, a stratified post-test-only cluster randomised controlled trial (cRCT) is used (Figure 1). In cRCTs, groups of people, rather than individuals, are randomised into an intervention and a control group. Because care pathways are complex interventions and they are developed for and will affect groups of people, a cluster randomised design is required to evaluate their effectiveness [24,25]. Each cluster consists of all individual members of the interprofessional team caring for patients hospitalized for COPD-exacerbation or PFF in a particular hospital. Stratified randomisation was used to assign the teams to an intervention group (using care pathways) and a control group (usual care). Interprofessional teams were randomised. COPD/PFF was used as blocking factor. To ensure that clusters in both arms are in balance, they are stratified for country-level, hospital type (teaching versus non teaching), size of hospitals (<600 and ≥600 beds), and annual volume of patients (<300 and ≥300 patients). Before the start of the randomisation process, random numbers were assigned to each cluster by a researcher not involved in the study, using the online available tool ‘Research Randomizer’ (http://www.randomizer.org). Next, the researcher randomly allocated the coded clusters to the intervention or control group using the same online tool. Through this, the randomisation process itself was fully concealed. Afterward, the research team and the participating teams were notified to which group they were allocated just before the start of the intervention. Each team randomised in the intervention group will implement the complex intervention for the development and implementation of a care pathway for COPD-exacerbation or PFF (see in more detail below). The teams randomised in the control group will not implement the complex intervention, and thus will provide usual care. The intervention teams will have about nine months to implement the complex intervention. After this time period, a summative evaluation will be performed in which performance on team indicators will be compared between the intervention and control group.

To enhance understanding on how the complex intervention, in case the care pathway, was actually implemented and under what conditions this implementation process was successful or not, the use of qualitative methods such as process evaluations alongside randomised controlled trials is advised [28]. This is because multiple behavioural and organisational factors will influence the extent in which the intervention can be integrated into everyday healthcare practice [29]. These factors will in turn determine their actual effectiveness. Therefore, the context in which the care pathway was developed and the implementation process will be evaluated using process measures during and after pathway-implementation in the intervention group.

### Inclusion and exclusion criteria

Inclusion on cluster level required the written agreement to participate from the hospital management, the medical head of the division, and the head nurse. In addition, they had to agree that they will not develop and implement a care pathway for COPD-exacerbation or PFF within the time frame of the study when randomised in the control group. Finally, the participating hospitals needed an expected volume of more than one hundred COPD-exacerbation or PFF patients annually. To have a comparable sample across all clusters, the following inclusion criteria on individual level are:

1. All professionals that according to the medical head of the division and head nurse are members of the interprofessional team caring for COPD-exacerbation or PFF patients from admission until discharge out of the acute hospital ward.

2. To be part of the interprofessional care team is further conceptualized as being part of the group of clinicians and staff who have a shared clinical purpose and direct care responsibilities for the respective patient group during the set time period.

3. All individual participants need to be member of the team during one specific week—chosen by the study coordinator and can be defined as an average week, with normal staff ratios—where patients are being followed for the two other trials of the EQCP project [23]; Because we want to study interprofessional teamwork, each cluster is asked to minimally include the orthopaedic surgeons/pneumologists, head nurse, nurses, physiotherapists, and social workers in their sample. Based on their own judgment, the medical head of the division, in consensus with the head nurse, can decide to include other professional groups in their sample.

4. In some hospitals COPD-exacerbation or PFF patients are being admitted at multiple nursing wards, *e.g.*, due to capacity problems or other organisational issues. If that is the case, then the clusters are asked to only include these team members that are working on the ward where the respective patient groups are being admitted most frequently.

Exclusion criteria are:

1. All team members that are not working (*e.g.*, on leave) during the chosen week.

2. All team members who are only temporarily part of the team (*e.g.*, student nurses in training).

### Sample size

**Figure 2:**
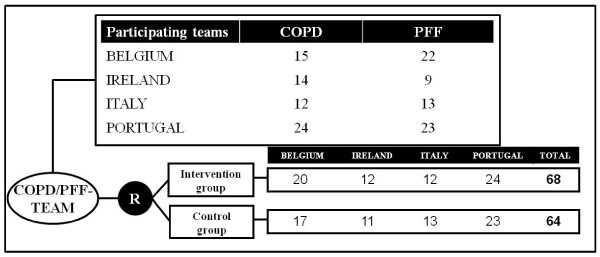
Participating teams in the EQCP-trial on teamwork.

In each of the four participating countries, hospitals were asked to participate by E-P-A in close cooperation with each national coordinator. In total, 132 teams (65 COPD teams and 67 PFF teams) agreed to participate (Figure 2). After the randomisation process, 68 teams were assigned to the intervention group (33 COPD teams and 35 PFF teams). Sixty-four teams were assigned to the control group (32 COPD teams and 32 PFF teams).

Sample size calculation in a cRCT is based on the expected improvement in the main outcome parameter [24,30]. Our research team decided to use the team process indicator ‘relational coordination’ as main outcome parameter for the EQCP-trial on teamwork. The concept of relational coordination (RC) describes a mutually reinforcing process of communicating and relating for the purpose of task integration [31]. The following criteria justify our choice: RC describes communication and relations within the type of teams we are focusing on; an international panel of scientific experts and hospital managers identified team communication and relations as highly relevant for follow-up as team indicators (Content Validity Indexes of 89% and 86%, respectively) [32], and both are dimensions measured with the concept of RC; Gittell *et al.* showed that there is a clear mediating relationship between RC and the performance effects of care pathways [10]; and systematic literature review showed a positive relation between care pathways and both team communication and relations [12].

To calculate the expected improvement, existing data available through the formative evaluation (see below) of the 20 participating Belgian clusters randomised in the intervention arm were used. This set contains the scores on RC of 362 participating individual team members. RC is scored on a scale from 1 (low RC) to 5 (high RC). The range of the average RC-scores on cluster level in the dataset was 2.9 to 3.9. The gap between the highest and lowest score was thus 1.34. We then identified 4 as cut-off score, because this is the average between our top score (3.9) and the top score (4.2) in the nine-hospital study of Gittell *et al.*[31]. In our sample, 30% of participants (107/362) scored higher than 4. If we take this actual gap as expected improvement, then we can expect that the percentage of participants scoring more than 4 would increase to 40% after care pathway implementation (30%*1.34). Based on a power of 80% and a significance level of 0.05 (two-sized), we would need up to 364 team members per arm to observe a difference of 10% (case = 40%; control = 30%) in number of participants scoring higher than 4 on RC. Given an inflation factor (IFF = 1.306) using the ICC out of two previous cRCTs on pathway-effectiveness by Panella *et al.* (ICC = 0.018) and the average sample size of the Belgian clusters in the intervention group during the formative evaluation (n = 17) [33,34], the required sample size increased to 475 team members per arm. Considering the average sample size of 17 team members per cluster, we need 28 clusters per arm.

### The complex intervention: care pathway implementation in the intervention arm

**Figure 3:**
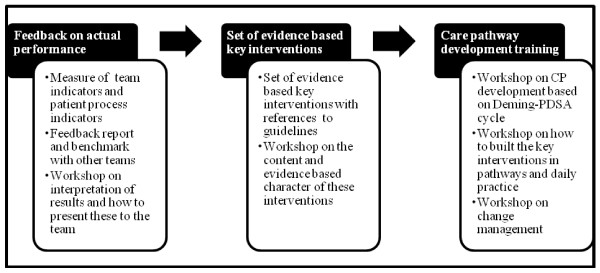
Active components of the EQCP-complex intervention.

**Figure 4:**
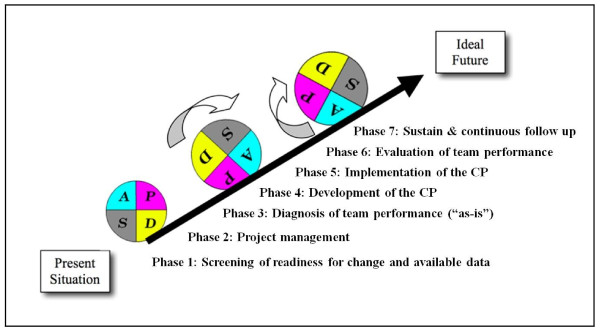
Care pathway implementation protocol based on the Deming-PlanDoStudyAct cycle.

The care pathway intervention contains three active components (Figure 3):

1. Formative evaluation of the teams’ performance before implementation: For this, a measure of the teams’ performance on team indicators and on patient process indicators is planned. The set of team indicators that will be used for this evaluation is described below. The patient process indicators are described in the research protocols of the other two EQCP trials [35,36]. These process indicators measure the teams’ compliance to the key interventions in the care process. Key interventions are those that, based on evidence-based medicine, need to be performed to guarantee high-quality care, and that thus will have significant impact on patient outcomes. An evaluation of performance to these process indicators will be performed for 20 consecutive patients. Each team will receive a feedback report with their own results, both on team and on patient level, benchmarked with the results of the other participating teams. Furthermore, a workshop per country will be organised on the interpretation of these results and on how these results should be presented to the teams. These workshops will be lead by an organizational psychologist (M.E.) and experts in care pathway development (K.V. and W.S.). All involved study coordinators will be invited. After a general introduction, the participants will be divided in small groups of five to help them interpret the results. The formative evaluation will help the teams to understand their actual performance, their points for improvement, and their actual level of organisation of the care process.

2. Evidence-based key interventions: Each team will receive a set of evidence-based key interventions for COPD-exacerbation or PFF. This set is based on an extensive literature review, Map of Medicine® (http://www.mapofmedicine.com), and on consensus by international clinical experts using a Delphi survey. The key interventions and outcomes include both in-hospital interventions and information for a safe discharge. A workshop will be organised on the content and evidence base of these interventions. These workshops will be lead by clinical experts in COPD and PFF. Next to the study coordinator, three key stakeholders of the participating teams will be invited. A teach-the-teacher based approach will be used.

3. Training in pathway development: Each study coordinator will be trained to develop the care pathway based on the findings of the formative evaluation. In the training workshop, a care pathway implementation protocol based on the Deming Plan-Do-Study-Act cycle and behavioural change strategies, which is generally accepted as the standard method for care pathway implementation, will be used (Figure 4) [37]. These one-day workshops will be led by experts in pathway development (W.S. and K.V.) and organizational and team change (M.E.). Again, a teach-the-teacher based approach will be used. Change will be further supported by giving the possibility to exchange best experiences among participating teams.

### Measures

The set of effect measures is described in Table 1. Teamwork is traditionally described using systems theory with an ‘input-process-output’ based approach [6]. This implies that effectiveness of teamwork is defined by a complex set of interactions between team inputs (context and structure of the team), team processes, and team outputs. Because we want to study the impact of care pathways on all facets of teamwork, we adopted this multifaceted ‘input-process-output’ approach in our choice of team indicators. The selection of measures is based on the international expert panel on relevant indicators to follow-up in care processes [32] and the systematic review of the relationship between care pathways and teamwork [12]. Hence, the research team made a substantiated decision on the measures based both on identified relevance to follow-up and expected effect of care pathways. As mentioned before, relational coordination is our primary effect measure. Next to the effect measures, a set of covariates will also be measured (Table 2). All team indicators will be analyzed on individual level, except for some team input indicators that will be measured on cluster level. These data will be collected with the use of both validated tools and structured questionnaires developed by the research team. The study coordinators will be trained in how all tools need to be completed. To guarantee anonymity, the team members will be asked to return the completed surveys to the study coordinators in a closed envelope. Afterward, these closed envelopes will be collected by a member of the research team.

**Table 1:** Set of effect measures

**Effect measures**	**Typology**	**Level of analysis**	**Description**	**Instrument**	**Respondents**
Team composition	Team input	Cluster level	The different professional groups of which the team is built up of.	Team membership list	Study coordinator
Team reflexivity	Team input	Cluster level	Frequency of follow up of the care process.	Structured questionnaire	Study coordinator
Team meetings	Team input	Cluster level	Frequency of team meetings.	Structured questionnaire	Study coordinator
			Number of different professional groups attending team meetings.		
Leadership structure	Team input	Cluster level	Leadership structure of the team: No team leader/1 team leader/shared leadership	Structured questionnaire	Study coordinator
Coordinating mechanisms	Team input	Cluster level	Use of guidelines and protocols, information systems, case management, interdisciplinary patient rounds, and interdisciplinary team meetings.	Structured questionnaire	Study coordinator
Dedicated team members	Team input	Individual level	If team members are exclusively dedicated to COPD of PFF care within the team or not.	Structured questionnaire	Team members
Perceived ‘teamness’	Team input	Individual level	To what extent is the team perceived by the team members as being a real interprofessional team?	Structured questionnaire (score 1–5)	Team members
Work environment	Team input	Individual level	To what extent is the work environment supportive for teamwork?	Structured questionnaire (score 1–5)	Team members
Management support	Team input	Individual level	To what extent is the hospital management supportive for teamwork?	Structured questionnaire (score 1–5)	Team members
Conflict management	Team process	Individual level	The quality of how conflicts are managed within the team.	Structured questionnaire (score 1–5)	Team members
Leadership quality	Team process	Individual level	Quality of leadership: general satisfaction with leader, leadership skills and effectiveness of leadership.	Structured questionnaire (score 1–5)	Team members
Team involvement	Team process	Individual level	How involved does each team member feel with the team?	Structured questionnaire (score 1–5)	Team members
Team climate for innovation	Team process	Individual level	- Team Vision: clarity of and commitment to objectives (4 items)	Team Climate Inventory[38]	Team members
			- Participative safety (4 items)		
			- Task orientation: emphasis on quality (3 items)		
			- Support for innovation (3 items)		
Relational coordination	Team process	Individual level	- Communication dimensions (4 items): frequent, timely, accurate and problem solving communication	Relational Coordination Survey[31]	Team members
			- Relationship dimensions (3 items): shared goals, shared knowledge and mutual respect		
Level of organised care	Team output	Individual level	Level of organisation of the care process:	Care Process Self Evaluation Tool[39]	Team members
			- Patient focused organisation (6 items)		
			- Coordination of CP (7 items)		
			- Communication with patient and family (4 items)		
			- Collaboration with primary care (3 items)		
			- Follow up of CP (9 items)		
Work Engagement	Team output	Individual level	Level of engagement to work:	Burn Out Inventory[40]	Team members
			- Emotional exhaustion (5 items)		
			- Mental detachment (5 items)		
			- Level of competence (5 items)		

**Table 2:** Set of covariates

**Covariates**	**Level of analysis**	**Description**	**Instrument**	**Respondents**
Randomisation group	Cluster level	Group in which the team was randomised.	Structured questionnaire	Study coordinator
Patient group	Cluster level	Patient group for which the care pathway is being developed.	Structured questionnaire	Study coordinator
Team size	Cluster level	Number of individuals in the team.	Team membership list	Study coordinator
Hospital characteristics	Cluster level	- Number of beds	Structured questionnaire	Study coordinator
		- Patient volume		
		- Teaching status		
		- Dedicated ward or not		
Team tenure	Cluster level	Average number of years that the team members are part of the team.	Structured questionnaire	Team members
Individual characteristics of the team member	Individual level	- Age and gender	Structured questionnaire	Team members
		- Professional group of team member		
		- % of working time		
		- Years of experience with the patient group		
		- Years of being a team member		
		- Has the team member a leading role within the team		

The process measures that will be used in the intervention group are described in Table 3. First, to measure the level of adherence to the complex intervention, the study coordinator will have to score to what extent the evidence-based key interventions were actually implemented. Second, to evaluate the implementation process, process follow-up files will be administered to the study coordinators at several time points during and after implementation. Furthermore, semi-structured interviews with key stakeholders of each cluster will be performed after implementation. We are planning to interview representatives of three groups of stakeholders: the study coordinator, hospital management, and key stakeholders of the interprofessional team. For each of these groups, a specific interview guide will be developed. Considering the large international sample, to enhance feasibility, the interviews will only be performed in a selection of clusters.

**Table 3:** Set of process measures

**Process measures**	**Typology**	**Description**	**Instrument**	**Respondents**
Adherence to the complex intervention	Implementation parameter	Process adherence parameter measuring the extent to which the evidence-based key interventions were actually implemented.	Process-follow-up files	Study coordinator
Project team composition	Implementation parameter	The composition of the project team: size and professional groups that are part of it.	Process-follow-up files	Study coordinator
Project satisfaction	Implementation parameter	Satisfaction with the implementation process.	Process-follow-up files	Study coordinator
Project meetings	Implementation parameter	Number of meetings the project team organised during the implementation process.	Process-follow-up files	Study coordinator
Facilitators and barriers	Implementation parameter	What facilitators and barriers were encountered during the implementation process of the care pathway?	Semi-structured interviews	Study coordinator and stakeholders of the management and team
Pathway effectiveness	Implementation parameter	How did the implementation of the care pathway affect patient care and teamwork?	Semi-structured interviews	Study coordinator and stakeholders of the management and team

### Statistical analysis

To analyze results, multilevel analysis will be performed. Common descriptive statistics (Fisher exact and Kruskal Wallis test for categorical and continuous variables, respectively) will be performed on the cluster level. The differences in the effect measures will be evaluated on team or individual level using respectively random-effects logistic or linear regression models, and accounting for the clustering effect. Statistical significance will be defined as a two-sided p-value <0.05. All analyses will be carried out using SAS 9.2 statistical software.

### Registration and ethical approval

The ethical approval is obtained on three levels: ethical approval by the ethical committee (EC) of the coordinating centre on country level: the EC of the University Hospitals of Leuven for Belgium (identifiers: B32220096036 and B32330096038), the EC of the National Committee of Data Protection for Portugal (identifiers: 6605/2011 and 6497/2011), the EC of AOU Maggiore della Carità di Novara for Italy (identifier: 625, 21/07/2011), and the EC of Mid-Western Regional Hospital, Limerick for Ireland; ethical approval with regard to the participation in the study is provided on cluster level, namely by the EC of each of the hospitals of the participating teams; and individual informed consent is sought from the team member with regard to the participation in surveys.

## Discussion

The EQCP study on teamwork is the first cRCT on the impact of care pathway implementation on interprofessional teamwork. As the World Health Organisation (WHO) World Alliance for Patient Safety points out, improving communication and coordination has to be priority number one for patient safety research and practice in developed countries [41]. Furthermore, team-directed implementation strategies for change in healthcare are still seldom studied [42]. Following Thomas *et al.*, we believe that care pathways, as being interprofessional quality improvement initiatives, can improve teamwork and install high-performing teams [21]. Through their use, care processes are organised, roles and tasks are standardized, clear team goals are set, and a team vision is built. This will lead to improved team communication and will install high quality interprofessional relations, which could in turn improve teamwork in all its facets.

Quality improvement initiatives as care pathways are strongly linked with behavioural change. Looking at the Theory of Planned Behaviour, effective behavioural change is guided by three kinds of considerations: normative, behavioural, and control beliefs [43]. With our complex intervention, containing three active building blocks, we expect to affect all three defining factors. The feedback on the actual performance should create normative beliefs and motivation for change. The set of evidence-based key interventions should create behavioural beliefs that comply with these interventions will actually improve patient outcomes and thus team performance. Finally, the training in care pathway development should install beliefs of control over factors that can facilitate or impede pathway implementation. Therefore, we are confident that with these three components, the teams will have the necessary tools to implement real change.

One strength of the study is the use of a high-quality cRCT design with multilevel analysis, as required to evaluate complex interventions affecting groups of people [24]. Second, we include in our design a realistic evaluation approach by using process evaluations [28]. Third, we study the impact on teamwork in all its facets, using an input-process-output approach. Fourth, the choice of team indicators to follow-up is based on extensive literature review and international expert panel. Fifth, we set clear inclusion criteria for team members, which will enhance the comparability of our clusters. On the contrary, we did give the medical head of the division and head nurse the opportunity to choose additional professional groups that are also members of their team according to them, because ‘team composition’ is one of our team input indicators. Because we expect that the intervention teams will evaluate the composition of their team, our hypothesis is that they will be composed of more professional groups than the control teams. Sixth, the international nature of the study, and because we both include COPD teams (medicine) as PFF teams (surgery), increases generalisability. Finally, with the large number of participating teams, we should have enough power to find significant effects.

A challenge for the study is that, although our complex intervention with its three building blocks is standardized, this does not imply that the implementation process will be fully under control. This is a key feature of complex interventions. The teams will have the ability to choose which key interventions they want to include or exclude. Organisational context and team conditions will influence the implementation process, and thus will have an impact on pathway-effectiveness [44]. Nonetheless, the implementation processes will be strictly followed-up using the Normalisation Process Model that will enable us to define active components and preferred implementation strategies [29]. Second, because teams involved in pathway development frequently are newly formed work groups and comprise different professionals with different professional backgrounds and cultures, these teams will have to struggle through the initial formative stages of group development [45]. Professional fragmentation and emerging team conflicts could therefore influence each professions’ response to the implementation of care pathways [46]. These specific conditions could thus influence our study results. Third, because care pathways are primarily used to improve patient processes, the focus during pathway development could center solely on compliance to the key interventions in the process and not on improving teamwork [21]. Fourth, because this is the first controlled trial that will study the impact of care pathways on relational coordination, no data out of literature were available to define the expected improvement. This increases uncertainty about our sample calculation. Fifth, non-response to surveys could influence the results. Sixth, results will be primarily based on self-reported measures, causing some limitations such as possible social desirability bias. Finally, the summative evaluation will occur at one time point only. Therefore, we will not be able to measure the sustainability of the effect of care pathways.

Results from our study will allow researchers and healthcare managers to draw conclusions on the interrelations and interactions between care pathway implementation and team inputs, processes, and outcomes. Through this, body of knowledge on the active components and facilitators of care pathway implementation will be further developed, and preferred implementation strategies can be defined. This should guide healthcare teams in actively improving the quality and safety of care processes.

## Abbreviations

EQCP-Study, European quality of care pathways study; cRCT, Cluster randomised controlled trial; PFF, Proximal femur fracture; COPD, Chronic obstructive pulmonary disease; IOM, Institute of medicine; AHRQ, Agency for healthcare and research quality; CP, Care pathways; EPA, European pathway association; RC, Relational coordination; EC, Ethical committee.

## Competing interests

The authors declare that they have no competing interests.

## Authors’ contributions

The study is coordinated by KV. All authors contributed in building the theoretical framework and methods. Writing of the study protocol was led by SD, with all authors commenting on drafts and approving the final version.

## References

[B1] Kohn LT Corrigan JM Donaldson MS To Err is human: building a safer health system 1999 National Academic Press, Washington DC 25077248

[B2] Committee on Quality of Health Care in America IoM Crossing the quality chasm: a new health system for the 21st century 2001 National Academy Press, Washington DC

[B3] Classen DC Resar R Griffin F Federico F Frankel T Kimmel N ‘Global trigger tool’ shows that adverse events in hospitals may be ten times greater than previously measured Health Aff 2011 30 4 581 589 10.1377/hlthaff.2011.0190 21471476

[B4] Xyrichis A Ream E Teamwork: a concept analysis J Adv Nurs 2008 61 2 232 241 10.1111/j.1365-2648.2007.04496.x 18186914

[B5] Salas E DiazGranados D Weaver SJ King H Does team training work? Principles for health care Acad Emerg Med 2008 15 11 1002 1009 10.1111/j.1553-2712.2008.00254.x 18828828

[B6] Baker DP Gustafson S Beaubien J Salas E Barach P Medical teamwork and patient safety: the evidence-based relation. Literature review 2005 AHRQ Publication No. 05–0053. Agency for Healthcare Research and Quality, Rockville

[B7] Salas E DiazGranados D Klein C Burke CS Stagl KC Goodwin GF Does team training improve team performance? A meta-analysis Hum Factors 2008 50 6 903 933 10.1518/001872008X375009 19292013

[B8] Sorbero ME Farley DO Mattke S Lovejoy S Outcomes measures for effective teamwork in inpatient care. RAND technical report TR-462-AHRQ 2008 RAND Corporation, Arlington

[B9] Allen D Gillen E Rixson L Systematic review of the effectiveness of integrated care pathways: what works, for whom, in which circumstances? Int J Evid Based Healthc 2009 7 61 74 10.1111/j.1744-1609.2009.00127.x 21631848

[B10] Gittell JH Coordinating mechanism in care provider groups: relational coordination as a mediator and input uncertainty as a moderator of performance effects Manag Sci 2002 48 11 1408 1426 10.1287/mnsc.48.11.1408.268

[B11] Gittell JH Seidner R Wimbush J A Relational model of how high-performance work systems work Organ Sci 2010 21 2 490 506 10.1287/orsc.1090.0446

[B12] Deneckere S Euwema M Van Herck P Lodewijckx C Panella M Sermeus W Care pathways lead to better teamwork: Results of a systematic review Soc Sci Med 2012 75 2 264 268 10.1016/j.socscimed.2012.02.060 22560883

[B13] Vanhaecht K Bollmann M Bower K Gallagher C Gardini A Guezo J Prevalence and use of clinical pathways in 23 countries - an international survey by the European pathway association E-P-A.org J Intgr Care Pathways 2006 10 28 34

[B14] McDonald KM Sundaram V Bravata DM Lewis R Lin N Kraft SA Closing the quality gap: a critical analysis of quality improvement strategies (Vol. 7: Care Coordination) 2007 Agency for Healthcare Research and Quality (US), Rockville Report No.: 04(07)-0051-7 20734531

[B15] Panella M Marchisio S Di SF Reducing clinical variations with clinical pathways: do pathways work? Int J Qual Health Care 2003 15 6 509 521 10.1093/intqhc/mzg057 14660534

[B16] Kwan J Sandercock P In-hospital care pathways for stroke Cochrane Database Syst Rev 2004 18 4 CD002924 15495038 10.1002/14651858.CD002924.pub2 PMC7003611

[B17] Rotter T Kugler J Koch R Gothe H Twork S van Oostrum JM A systematic review and meta-analysis of the effects of clinical pathways on length of stay, hospital costs and patient outcomes BMC Health Serv Res 2008 8 265 10.1186/1472-6963-8-265 19094244 PMC2632661

[B18] Barbieri A Vanhaecht K Van HP Sermeus W Faggiano F Marchisio S Effects of clinical pathways in the joint replacement: a meta-analysis BMC Med 2009 7 1 32 10.1186/1741-7015-7-32 19570193 PMC2715423

[B19] Evans-Lacko S Jarrett M McCrone P Thornicroft G Facilitators and barriers to implementing clinical care pathways BMC Health Serv Res 2010 10 182 10.1186/1472-6963-10-182 20584273 PMC2912894

[B20] Vanhaecht K De Witte K Sermeus W The care process organisation triangle: a framework to better understand how clinical pathways work J Intgr Care Pathways 2007 11 1 8

[B21] Thomas EJ Improving teamwork in healthcare: current approaches and the path forward BMJ Qual & Saf 2011 20 8 647 650 10.1136/bmjqs-2011-000117 21712372

[B22] Salas E King HB Rosen MA Improving teamwork and safety: Toward a practical systems approach, a commentary on Deneckere et al. Soc Sci Med 2012 75 6 986 989 10.1016/j.socscimed.2012.02.055 22627017

[B23] Vanhaecht K Sermeus W Peers J Deneckere S Lodewijckx C Leigheb F The European quality of care pathway (EQCP) study: history, project management & approach Int J Cancer 2010 14 2 52 56

[B24] Craig P Dieppe P Macintyre S Michie S Nazareth I Petticrew M Developing and evaluating complex interventions: the new Medical Research Council guidance BMJ 2008 337 a1655 10.1136/bmj.a1655 18824488 PMC2769032

[B25] Campbell NC Murray E Darbyshire J Emery J Farmer A Griffiths F Designing and evaluating complex interventions to improve health care BMJ 2007 334 7591 455 459 10.1136/bmj.39108.379965.BE 17332585 PMC1808182

[B26] Pawson R Tilley N Realistic Evaluation 1997 SAGE Publications Ltd, London

[B27] Berwick DM The science of improvement JAMA 2008 299 10 1182 1184 10.1001/jama.299.10.1182 18334694

[B28] Lewin S Glenton C Oxman AD Use of qualitative methods alongside randomised controlled trials of complex healthcare interventions: methodological study BMJ 2009 339 b3496 10.1136/bmj.b3496 19744976 PMC2741564

[B29] May C Finch T Mair F Ballini L Dowrick C Eccles M Understanding the implementation of complex interventions in health care: the normalization process model BMC Health Serv Res 2007 7 148 10.1186/1472-6963-7-148 17880693 PMC2089069

[B30] Campbell M Fitzpatrick R Haines A Kinmonth AL Sandercock P Spiegelhalter D Framework for design and evaluation of complex interventions to improve health BMJ 2000 321 7262 694 696 10.1136/bmj.321.7262.694 10987780 PMC1118564

[B31] Gittell JH Fairfield KM Bierbaum B Head W Jackson R Kelly M Impact of relational coordination on quality of care, postoperative pain and functioning, and length of stay: a nine-hospital study of surgical patients Medical Care 2000 38 8 807 819 10.1097/00005650-200008000-00005 10929993

[B32] Deneckere S Robyns N Vanhaecht K Euwema M Panella M Lodewijckx C Indicators for follow-up of multidisciplinary teamwork in care processes: results of an international expert panel Eval Health Prof 2011 34 3 258 277 10.1177/0163278710393736 21190951

[B33] Panella M Marchisio S Gardini A Di Stanislao F A cluster randomized controlled trial of a clinical pathway for hospital treatment of heart failure: study design and population BMC Health Serv Res 2007 7 179 10.1186/1472-6963-7-179 17986361 PMC2204000

[B34] Panella M Marchisio S Barbieri A Di SF A cluster randomized trial to assess the impact of clinical pathways for patients with stroke: rationale and design of the clinical pathways for effective and appropriate care study [NCT00673491] BMC Health Serv Res 2008 8 223 10.1186/1472-6963-8-223 18980664 PMC2585086

[B35] Vanhaecht K Sermeus W Peers J Lodewijckx C Deneckere S Leigheb F The impact of care pathways for exacerbation of chronic obstructive pulmonary disease: rationale and design of a cluster randomized controlled trial Trials 2010 11 111 10.1186/1745-6215-11-111 21092098 PMC3001422

[B36] Vanhaecht K Sermeus W Peers J Lodewijckx C Deneckere S Leigheb F The impact of care pathways for patients with proximal femur fracture: rationale and design of a cluster-randomized controlled trial BMC Health Serv Res 2012 12 124 10.1186/1472-6963-12-124 PMC3528433 22640531

[B37] Vanhaecht K Van Gerven E Deneckere S Lodewijckx C Janssen I VanZelm R The 7-phase method to design, implement and evaluate care pathways Int J Per Centered Med 2012 In Press

[B38] Anderson NR West MA Measuring climate for work group innovation: development and validation of the team climate inventory J Organ Behav 1998 19 235 258 10.1002/(SICI)1099-1379(199805)19:3<235::AID-JOB837>3.0.CO;2-C

[B39] Vanhaecht K De Witte K Depreitere R Van Zelm RT De Bleser L Proost K Development and validation of a care process self-evaluation tool (CPSET) Health Serv Manage Res 2007 20 189 202 10.1258/095148407781395964 17683658

[B40] Schaufeli WB Van Dierendonck D Utrechtse burnout schaal (UBOS): testhandleiding. [Utrecht burnout scale. Test manual] 2000 Harcourt Test Services, Amsterdam

[B41] Bates DW Larizgoitia I Prasopa-Plaizier N Jha AK Global priorities for patient safety research BMJ 2009 338 b1775 10.1136/bmj.b1775 19443552

[B42] Grol R Grimshaw J From best evidence to best practice: effective implementation of change in patients’ care Lancet 2003 362 9391 1225 1230 10.1016/S0140-6736(03)14546-1 14568747

[B43] Ajzen I Czasch C Flood MG From intentions to behaviour: Implementation intention, commitment, and conscientiousness J Appl Soc Psychol 2009 19 1356 1372

[B44] Ovretveit JC Shekelle PG Dy SM McDonald KM Hempel S Pronovost P How does context affect interventions to improve patient safety? An assessment of evidence from studies of five patient safety practices and proposals for research BMJ Quality & Safety 2011 20 7 604 610 10.1136/bmjqs.2010.047035 21493589

[B45] Tuckman BW Developmental sequence in small groups Psychological Bulletin 1965 63 384 399 14314073 10.1037/h0022100

[B46] Degeling P Maxwell S Kennedy J Coyle B Medicine, management, and modernisation: a ‘danse macabre’? BMJ 2003 326 7390 649 652 10.1136/bmj.326.7390.649 12649244 PMC1125544

